# Factors in Modulating the Potential Aromas of Oak Whisky Barrels: Origin, Toasting, and Charring

**DOI:** 10.3390/foods12234266

**Published:** 2023-11-25

**Authors:** Min Luo, Dongsheng Cui, Jin Li, Penghui Zhou, Changqing Duan, Yibin Lan, Guangfeng Wu

**Affiliations:** 1 Center for Viticulture and Enology, College of Food Science and Nutritional Engineering, China Agricultural University, Beijing 100083, China; LM05042023@163.com (M.L.); cuidongsheng@cau.edu.cn (D.C.); chqduan@cau.edu.cn (C.D.); lanyibin@cau.edu.cn (Y.L.); 2 Key Laboratory of Viticulture and Enology, Ministry of Agriculture and Rural Affairs, Beijing 100083, China; 3 Shandong Technology Innovation Center of Wine Grape and Wine, Yantai 264000, China; Lijin02@cofco.com (J.L.); zhoupenghui@cofco.com (P.Z.)

**Keywords:** origin, toasting intensity, charring degree, Chinese oak, oak–derived volatiles

## Abstract

In this study, the effects of origin (Chinese, France, and America), intensity of toasting, and degree of charring on the volatiles of oak whisky barrels were comprehensively investigated via liquid–liquid extraction–gas chromatography–mass spectrometry (LLE–GC–MS) combined with multivariate statistical analysis. Results of principal component analysis (PCA) showed that the main oak–derived volatiles in oak were more influenced by origin and toasting than by charring. French oak had a higher content of volatile compounds than the other two origins, and this difference decreased with toasting and charring. The process of toasting and charring was important for the release of volatile compounds from oak. The content of most oak–derived volatiles increased with deeper toasting intensity, and the degree of charring promoted or inhibited the release of oak–derived volatiles. The volatile components in oak blocks were affected by the two–factor interaction of toasting and charring. Continuing the process of the charring of oak at a certain level of toasting may have an enhancing or diminishing effect on the content of different volatile compounds, depending on the circumstances.

## 1. Introduction

Whisky is a popular drink in the Chinese market, especially among younger people. Barrel aging is an essential process of whisky production, as it improves the product’s stability, flavor complexity, and quality. Oak barrels play a very important role in this process, and the demand for oak barrels is very high. Most of the oak barrels currently on the market for whisky aging come from America and Europe. With the growth of the whisky industry in the Chinese market, the demand for Chinese oak is continuing to increase, with the aim of shaping Chinese–style whisky products to increase our competitiveness in the market. Several researchers have looked into the possibility of using indigenous Chinese oak to meet this demand [1]. Oak, also known as Quercus, is a genus of the family Fagaceae that grows in Europe, Asia, North America, and other regions. It is a common hardwood in the northern temperate zone [2]. There are more than 250 types of oak in the world, and three main types are commonly used in wine production: *Quercus sesiliflora* and *Q. robur* (from Europe), as well as *Q. alba* (from the United States) [3]. China has about 51 species of *Quercus* L. plants, among which *Q. mongolica* Fisch and *Q. liaotungensis* Koidz are the main ones that can be used for wine production. They are mainly distributed in the area spanning from Changbai Mountain to Daxinganling in Northeast China, as well as warm temperate deciduous forest areas. They cover an area of about 7,967,900 hm^2^ and account for 49.50% of the genus Quercus in China [4,5,6]. *Q. mongolica* is the predominant species in China, and its heartwood volatile compounds are similar to those of other species that have good oenological quality for barrel–making. Mongolian oak and Liaodong oak belong to the white oak class (*Q. fabri* Hance), and their wood structure differs greatly from that of American and French oak, but the strength and hardness of Mongolian oak and Liaodong oak are similar to those of American and French oak [7]. Moreover, these two domestic oak species are rich in cellulose (about 41–44%), hemicellulose (about 21–25%), lignin (about 24–30%), and tannin (about 9–16%) [3,8].

Greenwood is unsuitable for cooperage because it contains a lot of water (up to 70%) and its extractable compounds are not compatible with the goal of improving whisky quality, as it contains many bitter polyphenolic compounds (ellagitannins excess coumarins, etc.) and very few aromatic compounds [9]. In the manufacturing process of oak barrels for whisky aging, seasoning and toasting are the basic steps of barrel making; in addition, the process of charring is also included. These stages affect the structure and final chemical composition of the wood that will contact the whisky during aging. The wood loses water during seasoning, a process that is traditionally carried out in a natural, open–air drying environment, meaning that the wood is “air–dried”, but this process can also be artificially accelerated using kilns [10]. During this process, wood volatile compounds such as lactones, phenolic aldehydes, or volatile phenols show different behaviors; some of them increase their concentrations, while others decrease or show no significant variations. However, natural seasoning has a positive effect on the volatile composition of oak wood overall [11]. Toasting is one of the key steps in determining the physical and chemical compositions of woods, and it can potentially influence the chemical and sensory of the wine aged on barrels made from them [12]. Toasting causes physical and chemical modifications in the wood’s structure, such as softening due to the hydrolytic decomposition of hemicellulose and lignin, as well as the processes of pyrolysis and thermal hydrolysis. These processes are usually carried out using fire, natural gas, steam, and/or boiling water (or a combination of them) [10]. Different toasting levels are typically defined by target temperature and time reached on the staves. The staves can also be pre–soaked in water before toasting. This allows for higher temperatures without degrading biopolymers, lipids, and aromas [13]. Charring may be a complementary process to toasting and may promote the release of some volatile compounds or inhibit the synthesis of some volatile compounds. Charring can be achieved by an intense toasting fire that ignites the barrel or, alternatively, via the direct application of a natural gas flame, and each technique may yield different compounds [14]. Oak barrels generally go through a charring process to improve the sensory quality of the beverage through the degradation of biomacromolecules into smaller molecules, which can be incorporated into the beverage more easily [15]. An increased removal of sulfur compounds using charred wood has also been reported [16]. This process leads to the charring of some of the biopolymers, resulting in a layer of active charcoal on the inner surface of the barrel. This may help remove impurities that are present in the distillate aged in the barrel and catalyze other changes [17].

Previous studies have focused on American and European oak rather than Chinese oak, with great interest in toasting as an influencing factor and little attention being paid to charring. Most of the existing research on Chinese oak has focused on the effects of wine aging, and less emphasis has been placed on spirits such as whisky. The aim of this study was to investigate the differences in volatile compounds between oak of different origins, with different toasting and charring treatments, based on quantitative data obtained from LLE–GC–MS. In addition, to provide a basis for the production of oak and whisky with Chinese characteristics, the effects of toasting and charring on oak–derived volatiles were investigated.

## 2. Materials and Methods

### 2.1. Chemicals

Analytical–grade chemicals, including sodium chloride, sodium sulfate, and anhydrous copper sulfate, were purchased from Beijing Chemical Works (Beijing, China). Chromatographic–grade solvents, including ethanol and dichloromethane, were purchased from Honeywell (Marris Township, NJ, USA). The standards of volatile compounds used for quantification and C_6_–C_24_ *n*–alkanes were purchased from Sigma–Aldrich (St. Louis, MO, USA). The standard reagents we used include furfural, acetylfuran, 5–methyIfurfural, 2–furfurylalcohol, maltol, 5–hydroxymethylfurfural, *trans*–whisky lactone, methyl vanillate, coniferaldehyde, vanillin, acetovanilone, syringaldehyde, acetosyringone, 4–vinylguaiacol, *o*–cresol, *p*–cresol, *m*–cresol, guaiacol, 4–methylguaiacol, phenol, 4–ethylguaiacol, 4–propylguaiacol, eugenol, isoeugenol, and syringol.

### 2.2. Preparation of Oak Blocks

The oaks in this experiment were air–dried in open air under natural conditions for a period of 24 months. In this study, the oaks came from three origins: *Q. mongolica* came from China (C); *Q. alba* came from America (A); and *Q. robur* came from France (F). Oak samples with three intensities of toasting (light, medium, and heavy) and five levels of charring (0 (none), 1, 2, 3, and 4 (high)) were collected; two replicates were set for each sample. For each oak block, approximately 5 mm of the toasted or charred layer was scraped off the surface. Three toasting levels were carried out: light toasting (L), 185 ± 5 °C for 25 min; medium toasting (M), 195 ± 5 °C for 35 min; heavy toasting (H), 205 ± 5 °C for 45 min. Charring was carried out after toasting, and the five levels of charring were as follows: Charring degree 0 refers to a sample that was not charred, while charring degrees 1–4 represent sequential charring for 20, 25, 35, and 40 s with a 900–1200 °C flame. All oak samples in this study, regardless of origin, were toasted and charred. The raw oak (CK) without any treatment was considered as a control.

### 2.3. Extraction of Volatile Compounds

The extraction of volatile compounds from oak blocks was performed according to an ethanol solution extraction method that was optimized in the laboratory. First, 5.00 g of oak powder was weighed in a 250 mL conical flask, and 100 mL of 50% ethanol aqueous solution (EAS) was added. The flask was sealed and soaked for 24 h at 40 °C away from light. Then, the solution was diluted to 10% EAS. Next, 20 mL of the diluted solution was extracted with 5 mL of dichloromethane (DCM) and mixed with 10 µL of internal standard and 5 g of ammonium sulfate. The mixture was shaken for 5 min and centrifuged for 10 min at 8000 rpm, with a controlled temperature of 4 °C. The organic fractions were collected, and the extraction was repeated once more. After that, the organic fractions were combined, dried on sodium sulfate anhydrous (1.5 g), and concentrated to 1 mL under a nitrogen flush. Finally, the concentrated liquid was filtered through a 0.22 µm organic microporous filter membrane into a 2 mL injection vial. The experimental procedure described above was performed twice for each sample. The internal standards used were as follows: 3,4–dimethylphenol (2 g/L) for volatile phenols, *o–*vanillin (4 g/L) for phenolic aldehydes, and *γ–*hexalactone (4 g/L) for the remaining compounds.

### 2.4. Identification of Volatile Compounds in Oak Blocks

The identification of volatile compounds was carried out by comparing the obtained mass spectra and retention indices (RIs) with those of reference standards and compounds in the NIST 2011 mass spectrometry database through using the automatic mass spectral deconvolution and identification system (AMDIS). Compounds that lacked a standard were identified by comparing the retention indices (RIs) calculated using a C_6_–C_24_ *n*–alkane series under the same chromatographic conditions and mass spectra with compounds in the NIST 11 MS database.

### 2.5. Quantification of Volatile Compounds in Oak Blocks

The quantification of volatile compounds was performed using an Agilent 7890 gas chromatography equipped with an Agilent 5975 mass spectrometer (GC–MS system). A HP–INNOWAX capillary column (60 m × 0.25 mm id, 0.25 µm film thickness, J&W Scientific, Folsom, CA, USA) was used. The concentrated dichloromethane extract (1 μL) was injected in non–split mode. The oven temperature program was as follows: the initial temperature stood at 50 °C, and this was then increased to 127 °C at a rate of 7 °C/min and held for 3 min, increased to 170 °C at a rate of 4 °C/min, increased to 200 °C at a rate of 2 °C/min, and finally increased to 260 °C at a rate of 10 °C/min and held for 18 min. The temperatures of the ion source, transfer line, and quadrupole were 230, 250, and 150 °C, respectively. The mass spectrometer was operated in electron ionization (EI) mode at 70 eV and in full–scan mode, with data being collected from *m*/*z* 30 to 350.

The quantitation of volatile compounds was carried out according to the calibration curves of the aroma standards. A synthetic matrix containing 10% (*v*/*v*) ethanol/water solution was used. The aroma standards were diluted in 10 levels using the matrix solution, and data were collected under the same chromatographic conditions to obtain a calibration curve. The ratio of the peak area of the target compound to the peak area of the internal standard was calculated, and the proportional relationship between the peak area ratio and the concentration of the substance was utilized for quantification.

### 2.6. Statistical Analysis

Both a one–way and two–way analysis of variance (ANOVA) were performed using Duncan’s test at a significance level of *p* < 0.05. Both the one–way and two–way ANOVA and principal component analysis (PCA) were performed using the XLSTAT (2019) statistical software (Addinsoft, Paris, France).

## 3. Results and Discussion

In this study, 28 volatile compounds were identified in Chinese, American, and French oak, including 6 furfurans, 2 whisky lactones, 6 phenolic aldehydes, and 14 volatile phenols. The mass spectral information of the 28 representative oak aroma compounds identified via GC–MS, including the retention time, retention index, concentration range, and characteristic ions used to quantify of each aroma compound, is summarized in Table 1. The following discussion presents an exploratory analysis of the effects of origin, as well as that of degree of toasting and charring, on oak volatiles, as determined by carrying out a principal component analysis. Then, the effect of each factor and the interactions between the factors on oak volatiles are discussed.

### 3.1. Comparative Analysis of the Influence of Three Factors on the Volatile Compounds of the Oak Blocks

**Figure 1:**
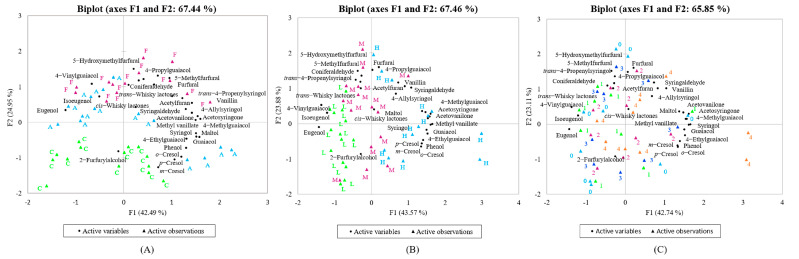
Biplot of the oak–derived volatile compounds’ PCA scores and loadings. The distribution of oak–derived volatile compounds in oaks according to origin (**A**), toasting intensity (**B**), and degree of charring (**C**). The letters C, A, and F stand for the countries of origin (China, America, and France), respectively; the letters L, M, and H stand for light, medium, and heavy toasting, respectively, and the numbers 0, 1, 2, 3, and 4 represent the degree of charring, with larger numbers representing deeper charring and 0 representing no charring.

The volatile compounds in the oak samples were analyzed via PCA for observations based on their origin, toasting intensity, and degree of charring, respectively (Figure 1), to visualize the differences between the samples and the importance of these three factors. A biplot of the oak-derived volatile compounds’ PCA scores and loadings are shown in Figure 1.

Figure 1A shows PCA score plots for observations based on oak origin. It can be seen that the second principal component (PC2) is almost successful in distinguishing oak samples of different origins, especially between Chinese and French oak, which are completely separated. American oak is in the middle, with a slight overlap with both Chinese and French oak. Figure 1B shows a PCA analysis of oak samples according to different degrees of toasting, and it can be found that the first principal component (PC1) can discriminate oak samples according to different degrees of toasting to some extent, but the degree of separation is not as obvious as for origin. Roughly speaking, the lightly toasted samples were on the negative side of F1; on the contrary, the heavily toasted samples were on the positive side, and the medium toasted ones were distributed in between. Figure 1C shows that it is difficult to distinguish the samples with different degrees of charring by means of principal components. Therefore, it can be concluded that origin, toasting intensity, and degree of charring have different effects on the volatile compounds in oak and that both origin and toasting intensity have a greater influence than charring. Mosedale et al. reported that the influence of the botanical species of oak on the oak–derived volatiles was undoubtedly an important factor, and the suitability of wood may vary not only between species but also between different geographical regions [18]. Jordão et al. also suggested that the physical properties and the structure of wood, such as the proportion of latewood to earlywood and the abundance of fibers, may influence heat conduction and reactions during heating [19].

### 3.2. Influence of Origins on the Volatile Compounds in the Oak Blocks

The origin of the oak influences the concentration of volatile compounds, which may depend on the environment, location, and species of the tree [19,20]. Table 2 presents the ANOVA of the quantitative results regarding the volatile compounds in oak samples of three different origins. The vast majority of the volatile compounds in the oak samples from different origins are significantly different, suggesting that these compounds are influenced by their origin. In general, the French oak blocks had higher levels of most volatile compounds than those from the other countries of origin, while the Chinese oak blocks had slightly lower levels of some volatile compounds. Generally, furfurans and phenolic aldehydes were abundant among the four main categories of volatile compounds. The effect of origin on the volatile compounds, according to their chemical categories, is discussed below.

#### 3.2.1. Furfurans

Origin, as a factor, was analyzed via a one–way ANOVA (Table 2). Regardless of origin, all oak blocks were rich in furfurans, especially furfural, 5–methylfurfural, and 5–hydroxymethylfurfural, as the main compounds in oak. Five of the six substances in the furfurans were significantly differentiated, and only maltol had no significant difference. Furfural, acetylfuran, 5–methylfurfural, and 5–hydroxymethylfurfural were all significantly higher in French oak than in the other two origins; this result is similar to that reported by Niu et al., who concluded that the content of furans was significantly higher in French oak than in Chinese oak [1]. Furfural, converted from the pentose unit of hemicellulose in wood, has a toasty odor and nutty odor, and furfural contributes significantly to the aroma during the aging process. It also forms 2–furaldehyde diethyl acetal by condensing with ethanol, which is an important volatile compound in oak [1,21]. 5–Methylfurfural and 5–hydroxymethylfurfural, obtainable from the conversion of hexose in oak cellulose, have a toasted and almond–like odor [8]. Furfural and 5–methylfurfural, which have been associated with “almond” and “toasted almond” aromas, are not thought to make significant contributions to wine aromas due to their relatively high threshold levels, but they may enhance the perception of whisky lactones [22]. Cadahía et al., concluded that 5–methylfurfural, 5–hydroxymethylfurfural, and furfural had higher mean values in French and American oak than in the other studied woods, which is consistent with the results of this paper [20]. 2–Furfurylalcohol, a reduced product of furfural, is generally found in relatively small amounts in oak. In this experiment, 2–furfurylalcohol was not dominant in content relative to other furans, but it was considered to be the most important compound of furfurans for its special caramelized, toasted aroma [19]. Because 2–furfurylalcohol was particularly abundant in Chinese oak, significantly higher than in the other two countries, there may be an aromatic contribution to the whisky aged in it, ultimately giving it a characteristic style.

#### 3.2.2. Whisky Lactones

Both *cis*– and *trans*–whisky lactones, which are important aroma compounds for oak, were detected in oak samples of all origins in this study. The *cis*– and *trans*–isomers of whisky lactones impart “coconut” and “oak” notes, giving elegant coconut and oak aromas to alcoholic beverages [23], and the richer the whisky lactones in oak, the more pronounced this flavor [24]. Whisky lactones had significant differences in their concentrations in the oak blocks from different origins, with the French oak blocks having the highest levels of *trans*–whisky lactones and American oak having the highest levels of *cis*–whisky lactones. The *cis*–whisky lactone content of Chinese oak was significantly lower than that of American oak and similar to that of French oak. This result is agreement with a previous study by Li et al. [4]. The ratio of *cis*/*trans* whisky lactone can be used to differentiate between French oak and American oak varieties. In this study, the ratio of whisky lactone isomers (*cis*/*trans*) was significantly different in the Chinese, American, and French oak blocks. This ratios were 31.19, 15.59, and 3.13 in the Chinese, American, and French oak blocks, respectively, confirming the usefulness of this parameter for distinguishing oak from different origins [25].

#### 3.2.3. Phenolic Aldehydes

The content of all phenolic aldehydes studied was abundant in all origins and was significantly higher in the French oak samples than in the samples from the other countries of origin, whereas the levels were similar in the Chinese and American oak samples (Table 2). This result may be related to the species of oak trees and the growing environment. Regardless of origin, the content of coniferaldehyde was abundant. Coniferaldehyde is formed by the depolymerization and oxidation of dimethoxy phenols such as coniferyl alcohol during the toasting process [26,27,28]. Its high content may be related to the high content of dimethoxy phenolic precursors in the lignin structure. Vanillin is an important aroma compound of oak that is abundant in oak of all origins and derived from the toasting process of oak and the hydrolysis of the lignin in the logs. It has a typical vanilla odor and a low sensory threshold in ethanol solutions (only 60 μg/L) [28]. Its vanilla odor and low threshold make it an important compound in oak that can contribute significantly to the aroma of whisky during aging. Syringaldehyde had the second highest levels among the phenolic aldehydes in oak from all origins (Table 2). Syringaldehyde has woody, vanilla, and bitter almond–like odors and is a characteristic aroma compound of oak [1]. These odors are also important and generally give a pleasant sensation, but they have a relatively high threshold (50,000 µg/L) and usually affect the wine aroma via superimposition or synergism [28]. Zhou et al. concluded that the concentrations of vanillin and syringaldehyde were highly correlated with the origin of oak and were higher in Chinese oak than in French and American oak at the same level of toasting [8]. However, the concentrations of vanillin and syringaldehyde in this study were abundant in oak from all three origins, which may be related to the soaking time during extraction.

#### 3.2.4. Volatile Phenols

Of the volatile phenols, all were significantly different between oak blocks of different various origins, with the exception of guaiacol, 4–ethylguaiacol, and eugenol. This indicates that the smoky and toasty aromas provided by guaiacol and 4–ethylguaiacol are minimally affected by origin [29]. The content of eugenol in the three origins did not differ significantly among the three origins, which is consistent with the results of a study by Li et al. [4]. French oak had significantly higher concentrations of 4–vinylguaiacol, 4–propylguaiacol, and isoeugenol than that of the other two countries. In addition, American oak wood had significantly higher levels of 4–allylsyringol and syringol. Small–molecule phenols (including phenol, *o–*cresol, *m–*cresol, and *p–*cresol) were generally not present at high levels and had higher concentrations in Chinese oak than in the other two origins. 4–Vinylguaiacol was also detected in this study and was produced by the decarboxylation of hydroxycinnamic acid (e.g., ferulic acid) during the aging process [30,31]. Its odor depends on the concentration and ranges from clove, nutty, and vanilla (less than 40 µg/kg) to undesirable odors such as leather, animal, stable, and pharmaceutical [29,30]. The concentration range determines the popularity of the whisky due to the variation in smell.

### 3.3. Influence of Toasting on Volatile Compounds in Oak Blocks

**Figure 2:**
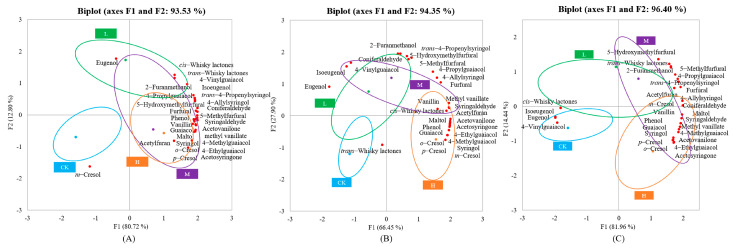
Biplot of the PCA–scores and –loadings of oak–derived volatile compounds at different intensities of toasting: Chinese oaks (**A**), American oaks (**B**), and French oaks (**C**). CK, L, M, and H stand for untoasted, light toasting, medium toasting, and heavy toasting, respectively.

To investigate the influence of different degrees of toasting on the volatile compounds and to reveal the possible differences between the different degrees of toasting, a principal component analysis was performed on untoasted oak and oak subjected to three different degrees of toasting (Figure 2). In this study, the samples were distinguishable at different toasting levels, especially between the untoasted and toasted samples, indicating that the degree of toasting had an influence on the volatile compounds in the oak. Although these oaks were collected from different regions, the distribution of samples with different degrees of toasting is consistent. Both the untoasted and lightly toasted oaks were on the negative side of F1. On the contrary, both the medium and heavily toasted oaks were on the positive side. In addition, a one–way analysis of variance (ANOVA) was performed on the content of volatile compounds in oak, with the degree of toasting as a factor. The majority of compounds showed an increase in values after toasting compared to those obtained in untoasted oak (Table S1), indicating that the volatile composition of oak wood is dependent on the temperature and duration of toasting. Combined with the results of the PCA (Figure 2), the toasting process could promote the accumulation of volatiles in the actual production process. The reason for this may be that heating operations during oak barrel manufacturing modify the macromolecular structure of wood, leading to the degradation of polysaccharides and polyphenols, the appearance of new compounds, and an increase in odorous volatiles such as furans and some phenols [19].

#### 3.3.1. Furfurans and Phenolic Aldehydes

All moderately and heavily toasted oak samples were on the positive side of F1 and had more compounds of furans and phenolic aldehydes (Figure 2), indicating that toasting may contribute to the formation of furfurans and phenolic aldehydes in oak. Phenolic aldehydes are produced by the thermal degradation of lignin in oak during the toasting process. Their content generally increased with the degree of toasting and had a strong vanilla odor [31]. This indicates that these aromas, i.e., the toasted and vanilla aromas, were more pronounced in oak with medium and higher degrees of toasting. Fernández de Simóna et al. concluded that coniferaldehyde and syringaldehyde were the compounds that were more correlated with toasting intensity and that their contents were significantly higher in heavily toasted oak than in oak with other degrees of toasting, similar to the findings of this study [9]. By conducting a one–way ANOVA, we found that all volatile compounds of furans and phenolic aldehydes were significantly different (*p* < 0.05) (Table S1), which also confirmed that the degree of toasting mainly influenced the formation of furfurans and phenolic aldehydes. Untoasted oak had the lowest levels of both furfurans and phenolic aldehydes, and the vast majority of furfurans and phenolic aldehydes were abundant in moderately and heavily toasted oak, being significantly higher than in untoasted and lightly toasted oak. The finding regarding the increase in furfuran content after toasting is consistent with a previous study by Jordão et al., who concluded that furfural and 5–methylfurfural were most affected by the toasting process [19]. There were, of course, exceptions: 2–furfurylalcohol and 5–hydroxymethylfurfural for Chinese and French oak were not only abundant in highly toasted oak but their content remained high in the lightly toasted samples, being significantly higher than in the untoasted oak samples.

#### 3.3.2. Whisky Lactones

The contents of *cis–* and *trans–*whisky lactones in the Chinese oak were on the positive side of F1 (Figure 2A). The *trans–*whisky lactone content in the American oak was on the negative side of F1, whereas the *cis–*whisky lactone content was on the positive side (Figure 2B). The distribution of *cis–* and *trans–*whisky lactones in the French oak was exactly the opposite of the distribution in the American oak (Figure 2C). This indicates that there is no common pattern to the effect of toasting on whisky lactones in oak from the three countries of origin. Considering the one–way ANOVA results (Table S1), it can be seen that the *trans–*whisky lactone content had no significant difference in samples with different levels of toasting. The *cis–*whisky lactone content was significantly different in Chinese and American oak at different toasting levels but not in French oak. *Cis–*whisky lactones in French oak were abundant in untoasted raw oak and oak of all toasting degrees. The findings of Fernández de Simón, B et al. are consistent with this study, as they argued that the *cis*– whisky lactone content was lower in toasted French oak than in untoasted wood, suggesting that the destruction of these components may occur during the toasting process in the cooperage [25]. The *cis–*whisky lactone content was significantly higher in lightly and heavily toasted Chinese oak than in the oak samples with other degrees of toasting, while for American oak, the content was significantly higher in oak subjected to medium and heavy toasting. The content of whisky lactones may depend on the oak itself and the oxidation of fatty acids during the toasting process [32]. Thus, the above results may indicate that the toasting process does not conceal the differences introduced by the factor of origin.

#### 3.3.3. Volatile Phenols

Most of the volatile phenols were on the positive half–axis of PC1 regardless of the origin of oak, indicating that most of the volatile phenols were abundant in oak subjected to medium and heavy toasting, and only some individual compounds were on the other side of F1 (Figure 2). Chira et al., concluded that volatile phenols are mainly derived from lignin degradation during the high–temperature toasting process, which gives a unique odor to various foods and beverages, including wine and whisky [29]. Volatile phenols such as eugenol were on the negative side of F1, suggesting that these substances may be abundant in untoasted and lightly toasted oak. Pierre–Louis Teissedre et al. also agreed that toasting may lead to eugenol degradation [33]. Except for *m*–cresol and *p*–cresol in French oak, the remaining volatile phenols were significantly different (Table S2), suggesting that these substances were significantly affected by the degree of toasting. Factoring in the results of our one–way ANOVA, the content of most volatile phenols increased with the degree of toasting, including guaiacol and syringol, which were significantly higher at high toasting levels than at low toasting degrees. Martínez–Gil et al. concluded that volatile phenols such as guaiacol and syringol were higher in toasted oak, which is consistent with the results of this study [34]. Individual compounds such as *m*–cresol in Chinese oak and eugenol in French oak were abundant at low toasting degrees.

### 3.4. Influence of Charring on the Volatile Compounds in the Oak Blocks

**Figure 3:**
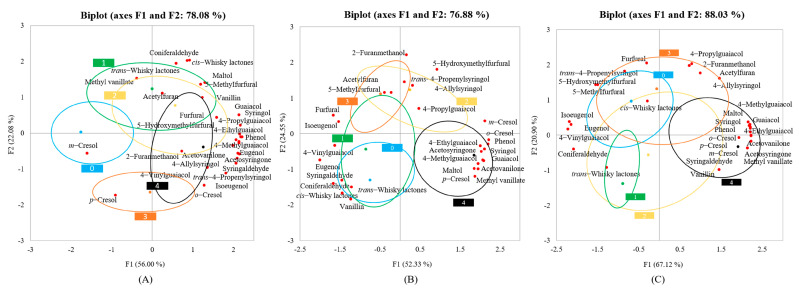
Biplot of the PCA–scores and –loadings of oak–derived volatile compounds at different degrees of charring: Chinese oak samples (**A**), American oak samples (**B**), and French oak samples (**C**). The numbers 0, 1, 2, 3, and 4 represent the degree of charring, with larger numbers representing deeper charring and 0 representing no charring.

In order to investigate the differences in volatile compounds in oak with different degrees of charring, a principal component analysis was performed on oak with five degrees of charring (Figure 3). In this study, the degree of separation between samples of different charring intensities was lower than the degree of toasting, suggesting consistency with the result above that the degree of toasting had a greater effect on the volatile compounds in oak than the degree of charring. The uncharred and low–charred oak samples were on the negative side of F1, whereas the highly charred oak samples were all on the positive side, with most of the volatile compounds (Figure 3). This shows that although the separation of oak samples at each degree of charring is not significant, the less charred samples could be separated from the highly charred samples, and this also indicates that most of the volatile phenols are more abundant in the highly charred oaks. This is consistent with the results of a study by Mosedale et al., who concluded that oak charring dramatically affects the volatile composition of oak wood and increases the levels of many barrel extractives [18]. A one–way analysis of variance (ANOVA) was also performed on the volatile compound contents of oak, considering the degree of charring as a factor. The content of most volatile compounds was higher in charred oak compared to uncharred oak (Table S2). Also considering the results of the PCA, it was found that the charring process promotes an increase in the content of most volatile compounds.

#### 3.4.1. Furfurans

It can be seen that furfurans were more abundant in oak with a high degree of charring (Figure 3), indicating that charring may contribute to the formation of furfurans in oak. Combined with a one–way ANOVA, except for maltol, the remaining five furfurans for Chinese oak were significantly different, mostly higher in the charred samples than in the uncharred ones (Table S2). In contrast, only a few furfurans were significantly different in American and French oak with different degrees of charring, indicating that, for furfurans, charring had the greatest effect on Chinese oak.

#### 3.4.2. Whisky Lactones

The whisky lactones discussed in the experiments were abundant in both the uncharred and low–charred samples (Table S2), and both *cis–* and *trans–*whisky lactones were significantly different in oak samples with different degrees of charring, suggesting that charring has an effect on whisky lactones. The contents of whisky lactones were significantly higher at lower charring levels than other charring levels in the Chinese and American oaks, and among the French oaks, uncharred oaks had the highest contents of whisky lactones. This indicates that the *cis–* and *trans–*whisky lactones are already high in raw and lightly charred oak and that the charring process reduces their content instead.

#### 3.4.3. Phenolic Aldehydes

Most of the phenolic aldehydes were distributed in the charred oak samples, indicating that charring has an effect on phenolic aldehydes and that charring can increase the content of phenolic aldehydes (Figure 3). With the exception of vanillin, all the other phenolic aldehydes in French oak were significantly different in samples with different degrees of charring, including syringaldehyde and acetosyringone, which were significantly higher in the most charred samples than in those subjected to other, lower levels of charring. In contrast, there were few phenolic aldehydes with significant differences in Chinese and American oak, three substances in Chinese oak, and only one in American oak, indicating that the effect of charring degree on phenolic aldehydes is most obvious in French oak.

#### 3.4.4. Volatile Phenols

Most of the volatile phenols were concentrated on the positive side of F1, which corresponds to the highly charred oak samples, showing that the highly charred samples contained a large amount of volatile phenols (Figure 3). Eugenol, isoeugenol, and 4–vinylguaiacol were significantly higher in uncharred oak than in charred oak for French oak (Table S2), indicating that the content of these substances is already high in the original French oak and that the charring process instead reduced the original content. All volatile phenols in French oak were significantly different between the different levels of charring; however, the volatile phenols in Chinese and American oak were less affected by charring than in French oak. There were no significant differences in small–molecule phenols (*o–*cresol, *p–*cresol, and *m–*cresol), guaiacol, and eugenol in Chinese and American oak.

### 3.5. Influence of the Interaction between Toasting and Charring on the Volatile Compounds in the Oak Blocks

Regardless of the results of the one–way ANOVA, all the compounds studied were significantly different under the two–factor effect of toasting and charring (Table S3). Some substances were not significantly different in the single factor of toasting but had highly significant differences in the interaction between toasting and charring, such as the *trans–*whisky lactones in Chinese and American oak, as well as the *cis–*/*trans–*whisky lactones, *p–*cresol, and *m–*cresol in French oak, indicating that toasting has a lesser effect on these compounds than charring. In contrast, some aroma compounds were not significantly different under the single factor of charring but had highly significant differences under the interaction, such as maltol, vanillin, guaiacol, and eugenol in Chinese and American oak, as well as furfural, 5–methylfurfural, 5–hydroxymethylfurfural, and vanillin in French oak, indicating that toasting has a greater effect on them than charring. Eugenol was abundant in untreated or lightly treated oak samples of all three origins, indicating that eugenol is abundant in raw oak and that either toasting or charring reduces its content.

Continuing the process of charring oak at a certain level of toasting may have an enhancing or diminishing effect on the content of different volatile compounds, depending on the circumstances. It is worth mentioning that the content of volatile compounds was higher in charred samples of Chinese oak under light and medium toasting, while under heavily toasting, it was more abundant in uncharred oak and significantly higher than in charred oak. This suggests that within a certain range, charring has an effect that promotes the production of volatile compounds just as much as toasting does, and beyond this range, it may reduce the content of volatile compounds. Although the effect of charring is not as great as that of toasting, this process cannot be ignored. In contrast to Chinese oak, the content of most volatile compounds in American and French oak at the heavily toasted with the highest degree of charring was significantly higher than in the other samples. The reason for this may be that the content of these volatile compounds is released more completely as the toasting and charring process progresses. In contrast, volatile compounds such as 4–vinylguaiacol and isoeugenol were very abundant in French raw oak, with their contents being significantly higher than in other toasted and charred oaks, indicating that these compounds may be broken down after the toasting and charring process or that toasting and charring may inhibit their production.

## 4. Conclusions

In this study, origin and toasting were found to have a greater influence on the volatiles than the charring process. Most of the volatile compounds were higher in French oak than in the oak of the other countries of origin, but the difference caused by origin diminished due to toasting and charring. 2–Furfurylalcohol and some small molecules of phenolic aldehydes were abundant in Chinese oak compared to oak from other origins. In general, the concentration of most oak–derived volatiles increased with a higher toasting intensity, and the contents of oak–derived volatiles were dependent on the temperature and duration of toasting. Charring is a continuation of the toasting process, and despite the effect of charring not being as great as that of toasting, this process cannot be ignored. The process of charring promoted or inhibited the release of oak–derived volatiles depending on the circumstances. For a small number of substances, toasting and charring may reduce their levels, probably because toasting and charring break down these substances in the original oak. The two–factor interaction of toasting and charring had an effect on the contents of the oak–derived volatiles studied. For the volatile compounds in oak, charring can be complementary when toasting intensity is not reached, while charring may be inhibitory when toasting is sufficient. In Chinese oak, charring increased the content of volatile compounds in light– and medium–toasted oak, while in heavily toasted oak, the content of volatile compounds was lower in highly charred oak. Oak after charring also has an adsorptive effect in the aging of whisky, and the influence of charring on whisky aroma is worthy of further investigation.

For oak barrel manufacturing, toasting and charring have to work in tandem with each other to facilitate optimal oak barrels in order to offer the possibility of aging whisky with Chinese characteristics. The style of whisky production and the duration of aging should be considered when oak barrels of different origins with different degrees of toasting and charring are used due to their great variation in terms of volatile compounds. Additionally, how the aging of different oak barrels affects the volatile profiles and sensory characteristics of the final whisky product will be further investigated in a subsequent study.

## Figures and Tables

**Table 1:** Information of 28 oak–derived volatiles identified using the LLE–GC–MS method.

Oak–Derived Volatile Compounds	CAS	RT (min)	RIs ^a^	LRIs ^b^	Qualitative Method ^c^	Ion (*m*/*z*)	Calibration Curve	**Concentration Range** **(μg/L)**
**Furfurans**								
Furfural	98–01–1	16.42	1479	1461	SD	96	y = 1074.90x − 18.73	13.863~7098
Acetylfuran	1192–62–7	17.61	1521	1499	SD	95	y = 484.18x − 6.931	3.609~1848
5–Methylfurfural	620–02–0	19.57	1588	1570	SD	110	y = 915.76x − 12.61	6.879~3522
2–Furfurylalcohol	98–00–0	21.75	1669	1660	SD	98	y = 8595.90x − 5.938	7.008~3588
Maltol	118–71–8	31.06	1974	1969	SD	126	y = 3192.50x + 4.934	2.061~1055
5–Hydroxymethylfurfural	67–47–0	46.45	2508	2496	SD	97	y = 6469.50x − 14.06	13.467~6895
**Whisky lactones**								
*trans–*Whisky lactone	39212–23–2	28.70	1900	1968	SD	99	y = 1076x − 10.69	5.695~2916
*cis–*Whisky lactone	55013–32–6	30.96	1969	1957	D	99	y = 1076x − 10.69	5.695~2916
**Phenolic aldehydes**								
Methyl vanillate	3943–74–6	48.77	2644	2600	SD	151	y = 198.41x + 1.23	2.617~1340
Coniferaldehyde	458–36–6	63.63	3031	3038	SD	178	y = 1971.90x + 4.91	2.002~1025
Vanillin	121–33–5	48.01	2581	2568	SD	151	y = 299.07x + 8.54	13.926~7130
Acetovanilone	498–02–2	49.81	2654	2640	SD	151	y = 217.37x + 3.09	4.697~2405
Syringaldehyde	134–96–3	57.31	2962	2919	SD	182	y = 422.31x − 0.43	3.164~1620
Acetosyringone	2478–38–8	58.63	3246	3244	SD	181	y = 270.97x − 0.12	1.230~630
**Volatile phenols**								
4–Allylsyringol	6627–88–9	47.19	2549	2561	D	194	y = 339.40x − 2.76	0.723~370
*trans*–4–Propenylsyringol	20675–95–0	52.12	1701	1704	D	194	y = 339.40x − 2.76	0.723~370
4–Vinylguaiacol	7786–61–0	38.85	2208	2188	SD	135	y = 226.34x − 1.17	9.766~5000
*o*–Cresol	95–48–7	32.01	2016	2008	SD	108	y = 590.09x + 0.30	0.840~430
*p*–Cresol	106–44–5	32.14	2086	2080	SD	107	y = 769.50x − 0.29	0.771~395
*m*–Cresol	108–39–4	35.37	2102	2091	SD	108	y = 425.52x − 0.20	0.820~420
Guaiacol	90–05–1	27.65	1866	1861	SD	109	y = 226.34x − 1.17	1.309~670
4–Methylguaiacol	93–51–6	30.62	1968	1956	SD	138	y = 406.97x − 1.30	0.586~300
Phenol	108–95–2	32.28	1019	2000	SD	94	y = 243.29x − 0.57	0.781~400
4–Ethylguaiacol	2785–89–9	33.02	2034	2032	SD	137	y = 187.09x − 0.19	0.742~380
4–Propylguaiacol	2785–87–7	35.71	2159	2103	SD	137	y = 99.58x + 0.13	0.703~360
Eugenol	97–53–0	38.21	2171	2169	SD	164	y = 401.26x + 0.09	0.977~500
Isoeugenol	97–54–1	43.18	2357	2318	SD	164	y = 1192.30x − 8.99	4.043~2070
Syringol	91–10–1	41.09	2269	2273	SD	154	y = 339.40x − 2.76	0.723~370

^a^ RIs: Retention indices on HP–INNOWAX capillary column. ^b^ LRIs: Linear retention indices on standard polar column referenced from NIST 11 database. ^c^ Qualitative way of compounds. S: Identification based on retention indices and mass spectrometry compared with standard compound; D: Identification based on retention index and mass spectrometry compared with NIST database.

**Table 2:** Analysis of variance (ANOVA) for the content of oak–derived volatiles in oak blocks of different origins ^a^ (μg/g).

Compound	*p* Value	Country		
		C	A	F
**Furfurans**				
Furfural	***	857.17 c ^b^	2048.68 b	2748.46 a
Acetylfuran	***	0.77 c	3.20 b	5.35 a
5–MethyIfurfural	***	144.73 c	233.87 b	374.16 a
2–Furfurylalcohol	***	65.69 a	29.08 b	21.68 c
Maltol	=	67.27	80.49	69.49
5–Hydroxymethylfurfural	***	464.81 c	1035.14 b	1585.18 a
**Whisky lactones**				
*trans–*Whisky lactone	***	3.22 c	16.06 b	46.24 a
*cis–*Whisky lactone	***	100.30 c	250.41 a	144.86 b
**Phenolic aldehydes**				
Methyl vanillate	*	2.49 b	2.95 ab	3.40 a
Coniferaldehyde	***	1545.47 b	1244.33 b	2435.83 a
Vanillin	***	130.63 b	131.35 b	208.42 a
Acetovanilone	***	20.38 b	21.78 b	34.04 a
Syringaldehyde	***	539.38 c	758.19 b	927.29 a
Acetosyringone	***	52.30 c	83.87 b	113.15 a
**Volatile phenols**				
4–Allylsyringol	**	20.51 b	25.99 a	22.77 ab
*trans–*4–Propenylsyringol	***	91.95 b	150.92 a	139.03 a
4–Vinylguaiacol	***	7.60 b	7.45 b	9.25 a
*o–*Cresol	***	1.41 a	1.04 a	0.56 b
*p–*Cresol	***	1.15 a	0.84 a	0.22 b
*m–*Cresol	***	0.71 a	0.45 b	0.18 c
Guaiacol	=	8.35	11.41	9.30
4–Methylguaiacol	**	6.39 b	9.45 a	9.71 a
Phenol	**	2.05 a	1.48 b	1.44 b
4–Ethylguaiacol	=	3.49	4.46	3.76
4–Propylguaiacol	***	8.97 c	12.63 b	17.20 a
Eugenol	=	13.35	13.10	12.69
Isoeugenol	*	201.61 ab	187.21 b	218.52 a
Syringol	*	51.26 b	89.79 a	62.86 b

^a^ Significant difference in the concentrations of oak–derived volatiles between Chinese oaks, American oaks, and French oaks are indicated by *, *p* < 0.05; **, *p* < 0.01; and ***, *p* < 0.001. ^b^ Different letters in the same row indicate significant differences in the concentrations of oak–derived volatiles between Chinese oaks, American oaks, and French oaks at the level of *p* < 0.05. C, Chinese oaks; A, American oaks; F, French oaks; =, no significant difference.

## Data Availability

Data are contained within the article.

## Supplementary Materials

The following supporting information can be downloaded at: https://www.mdpi.com/article/10.3390/foods12234266/s1, Table S1: Analysis of variance (ANOVA) for the content of aroma substances in oak with different intensities of toasting (μg/g); Table S2: Analysis of variance (ANOVA) for the content of aroma substances in oak with different levels of charring (μg/g); Table S3: Two–way analysis of variance (ANOVA) for the content of aroma substances in oak with different degrees of toasting and charring (μg/g).

## References

[1] Niu J.M., Zhang B., Wu J.D., An S.D., Ma C.L., Shi X., Han S.Y. (2021). Analysis of volatile components in the heartwood of *Quercus liaotungensis* Koidz and *Q. mongolica* Fisch. Food Sci..

[2] Rudnitskaya A., Schmidtke L.M., Reis A., Domingues M.R.M., Delgadillo I., Debus B., Kirsanov D., Legin A. (2017). Measurements of the effects of wine maceration with oak chips using an electronic tongue. Food Chem..

[3] Ma W.P., Sun Y., Ni Z.J., Wang W., Song C.B. (2016). Comparison of physiochemical properties of oaks from Liupan Mountain regions of Ningxia and European and American oaks. Liquor.-Mak. Sci. Technol..

[4] Li L.X., Li J.M., Zhao H., Jiang W.G., Yu Y., Sheng Z.Y. (2016). Difference in main compositions between domestic and European/American oak. Liquor.-Mak. Sci. Technol..

[5] Wang J.X. (2017). Current status and development trend of China’s oak resource utilization technology research. Prot. For. Sci. Technol..

[6] Li Y.Q. (2011). Resource Investigation and Superior Germplasm Resources Selection of Woody Energy Plants *Quercus mongolica* Fisch and *Quercus liaotungensis* Koidz. Unpublished. Doctoral Dissertation.

[7] Chen X.L. (2012). Study on the Changes of Chemical Compounds in Cabernet Sauvignon Dry Red Wine Storage.

[8] Zhou S., Xu Y., Fan W.L., Li J.M., Yu Y., Jiang W.G., Li L.X. (2012). Analysis on volatile compounds in oak chips with medium toasting level using liquid-liquid extraction coupled with gas chromatography-mass spectrometry. Food Ferment. Ind..

[9] De Simón B.F., Cadahia E., del Alamo M., Nevares I. (2010). Effect of size, seasoning and toasting in the volatile compounds in toasted oak wood and in a red wine treated with them. Anal. Chim. Acta.

[10] Casassa L.F., Ceja G.M., Vega-Osorno A., du Fresne F., Llodra D. (2021). Detailed chemical composition of Cabernet Sauvignon wines aged in French oak barrels coopered with three different stave bending techniques. Food Chem..

[11] Doussot F., De Jéso B., Quideau S., Pardon P. (2002). Extractives Content in Cooperage Oak Wood during Natural Seasoning and Toasting; Influence of Tree Species, Geographic Location, and Single-Tree Effects. J. Agric. Food Chem..

[12] Navarro M., Kontoudakis N., Gomez-Alonso S., Garcia-Romero E., Canals J.M., Hermosin-Gutierrez I., Zamora F. (2016). Influence of the botanical origin and toasting level on the ellagitannin content of wines aged in new and used oak barrels. Food Res. Int..

[13] Hale M.D., McCafferty K., Larmie E., Newton J., Swan J.S. (1999). The influence of oak seasoning and toasting parameters on the composition and quality of wine. Am. J. Enol. Vitic..

[14] Gollihue J., Pook V.G., DeBolt S. (2021). Sources of variation in bourbon whiskey barrels: A review. J. Inst. Brew..

[15] Barbosa R.B., Santiago W.D., Alvarenga G.F., da Silva Oliveira R.E., Ferreira V.R.F., Nelson D.L., das Graças Cardoso M. (2022). Physical–Chemical Profile and Quantification of Phenolic Compounds and Polycyclic Aromatic Hydrocarbons in Cachaça Samples Aged in Oak (*Quercus* sp.) Barrels with Different Heat Treatments. Food Bioproc. Tech..

[16] Clyne J., Conner J.M., Paterson A., Piggott J.R. (1993). The effect of cask charring on Scotch whisky maturation. Int. J. Food Sci. Technol..

[17] Lee K.Y.M., Paterson A., Piggott J.R., Richardson G.D. (2001). Origins of Flavour in Whiskies and a Revised Flavour Wheel: A Review. J. Inst. Brew..

[18] Mosedale J.R. (1995). Effects of oak wood on the maturation of alcoholic beverages with particular reference to whisky. Forestry.

[19] Jordão A.M., Ricardo-da-Silva J.M., Laureano O., Adams A., Demyttenaere J., Verhé R., De Kimpe N. (2006). Volatile composition analysis by solid-phase microextraction applied to oak wood used in cooperage (*Quercus pyrenaica* and *Quercus petraea*): Effect of botanical species and toasting process. J. Wood Sci..

[20] Cadahía E., Muñoz L., de Simón B.F., García-Vallejo M.C. (2001). Changes in Low Molecular Weight Phenolic Compounds in Spanish, French, and American Oak Woods during Natural Seasoning and Toasting. J. Agric. Food Chem..

[21] Garcia R., Soares B., Dias C.B., Freitas A.M.C., Cabrita M.J. (2012). Phenolic and furanic compounds of Portuguese chestnut and French, American and Portuguese oak wood chips. Eur. Food Res. Technol..

[22] Spillman P.J., Sefton M.A., Gawel R. (2004). The contribution of volatile compounds derived during oak barrel maturation to the aroma of a Chardonnay and Cabernet Sauvignon wine. Aust. J. Grape Wine Res..

[23] Garde-Cerdán T., Lorenzo C., Carot J.M., Jabaloyes J.M., Esteve M.D., Salinas M.R. (2008). Statistical differentiation of wines of different geographic origin and aged in barrel according to some volatile components and ethylphenols. Food Chem..

[24] Li S., Crump A.M., Grbin P.R., Cozzolino D., Warren P., Hayasaka Y., Wilkinson K.L. (2015). Aroma Potential of Oak Battens Prepared from Decommissioned Oak Barrels. J. Agric. Food Chem..

[25] Brı´gida F.n.d.S.n., Estrella C., Jerzy J. (2003). Volatile Compounds in a Spanish Red Wine Aged in Barrels Made of Spanish, French, and American Oak Wood. J. Agric. Food Chem..

[26] Sanz M., de Simón B.F., Cadahía E., Esteruelas E., Muñoz A.M., Hernández T., Estrella I., Pinto E. (2012). LC-DAD/ESI-MS/MS study of phenolic compounds in ash (*Fraxinus excelsior* L. and *F. americana* L.) heartwood. Effect of toasting intensity at cooperage. J. Mass. Spectrom..

[27] Boerjan W., Ralph J., Baucher M. (2003). Lignin Biosynthesis. Annu. Rev. Plant Biol..

[28] Cerdán T.G., Goñi D.T., Azpilicueta C.A. (2004). Accumulation of volatile compounds during ageing of two red wines with different composition. J. Food Eng..

[29] Chira K., Teissedre P.-L. (2013). Extraction of oak volatiles and ellagitannins compounds and sensory profile of wine aged with French winewoods subjected to different toasting methods: Behaviour during storage. Food Chem..

[30] Spillman P.J., Sefton M.A., Gawel R. (2004). The effect of oak wood source, location of seasoning and coopering on the composition of volatile compounds in oak-matured wines. Aust. J. Grape Wine Res..

[31] García-Carpintero E.G., Gallego M.G., Sánchez-Palomo E., Viñas M.G. (2012). Impact of alternative technique to ageing using oak chips in alcoholic or in malolactic fermentation on volatile and sensory composition of red wines. Food Chem..

[32] Bo Z., Fei H., Jian C., Yunhe W., Changqing D., Shunyu H. (2017). Effect of Toasting Intensity and Wood Grain on Polyphenolic Compounds and Aroma Components in Oak (*Quercus petraea*) Heartwood. Food Sci..

[33] Chira K., Teissedre P.-L. (2015). Chemical and sensory evaluation of wine matured in oak barrel: Effect of oak species involved and toasting process. Eur. Food Res. Technol..

[34] Martínez-Gil A.M., del Alamo-Sanza M., del Barrio-Galán R., Nevares I. (2022). Alternative Woods in Oenology: Volatile Compounds Characterisation of Woods with Respect to Traditional Oak and Effect on Aroma in Wine, a Review. Appl. Sci..

